# New Perspectives on the Use of Phytochemicals as an Emergent Strategy to Control Bacterial Infections Including Biofilms

**DOI:** 10.3390/molecules21070877

**Published:** 2016-07-05

**Authors:** Anabela Borges, Ana Cristina Abreu, Carla Dias, Maria José Saavedra, Fernanda Borges, Manuel Simões

**Affiliations:** 1 LEPABE, Department of Chemical Engineering, Faculty of Engineering, University of Porto, Rua Dr. Roberto Frias, s/n, Porto 4200-465, Portugal; anabreu@fe.up.pt (A.C.A.); cdias@utad.pt (C.D.); 2 CIQUP, Department of Chemistry and Biochemistry, Faculty of Sciences, University of Porto, Rua do Campo Alegre, s/n, Porto 4169-007, Portugal; fborges@fc.up.pt; 3 CECAV-Veterinary and Animal Science Research Center, Department of Veterinary Science, University of Trás-os-Montes e Alto Douro, Apartado 1013, Vila Real 5001-801, Portugal; saavedra@utad.pt

**Keywords:** antibiotic adjuvants, antibiofilm strategies, multidrug resistance, efflux pump inhibition, metal chelators, plant compounds, quorum sensing inhibition

## Abstract

The majority of current infectious diseases are almost untreatable by conventional antibiotic therapy given the advent of multidrug-resistant bacteria. The degree of severity and the persistence of infections are worsened when microorganisms form biofilms. Therefore, efforts are being applied to develop new drugs not as vulnerable as the current ones to bacterial resistance mechanisms, and also able to target bacteria in biofilms. Natural products, especially those obtained from plants, have proven to be outstanding compounds with unique properties, making them perfect candidates for these much-needed therapeutics. This review presents the current knowledge on the potentialities of plant products as antibiotic adjuvants to restore the therapeutic activity of drugs. Further, the difficulties associated with the use of the existing antibiotics in the treatment of biofilm-related infections are described. To counteract the biofilm resistance problems, innovative strategies are suggested based on literature data. Among the proposed strategies, the use of phytochemicals to inhibit or eradicate biofilms is highlighted. An overview on the use of phytochemicals to interfere with bacterial quorum sensing (QS) signaling pathways and underlying phenotypes is provided. The use of phytochemicals as chelating agents and efflux pump inhibitors is also reviewed.

## 1. Introduction

In their ecosystem, plants are continuously exposed to a wide range of environmental stresses and hostile conditions. Stress factors affecting plant fitness include environmental (abiotic) factors, such as nutrient deficiency, hypoxia/anoxia, drought, salinity, lack of oxygen, adverse temperature fluctuations, high light intensity, and also those factors derived from anthropogenic activities, such as pesticides, pollutants and increased UV radiation [1,2]. Furthermore, several living (biotic) factors are also stress-inducing factors, including bacteria, fungi, viruses, nematodes, insects and herbivore pests [3]. Plants have faced most of their attackers for more than 350 million years. This allowed plants to co-evolve with their natural enemies in a reciprocal evolutionary interaction and to learn how to resist their attacks. Although lacking mobile defender cells and a somatic adaptive immune system comparable to that of animals [4], plants have the ability to recognize pathogen signals or elicitors and activate immune responses through the reinforcement of the cell wall, the biosynthesis of lytic enzymes, and the production of secondary metabolites and pathogenesis-related proteins [5]. Owing to their sessile lifestyle and this evolutionary arms race, plants have evolved a stunning broad array of chemical defenses formerly referred to as secondary metabolites. These compounds can be either constitutive, stored as inactive forms, or inducible in response to pathogen attack [6]. The former are known as phytoanticipins and the latter as phytoalexins. Phytoanticipins (including saponins, cyanogenic glycosides and glucosinolates) are present in the plant before microorganism attack, or produced after infection from pre-existing precursors [7]. Some phytoanticipins are found at the plant surface while others are present in vacuoles or organelles and are released through a hydrolyzing enzyme after pathogen challenge [8]. Phytoalexins (including terpenoids, glycosteroids, flavonoids and polyphenols) are small molecules (molecular weight < 500) which are both synthesized and accumulated in the plant after the recognition of elicitors derived from exposure to attackers [9]. Besides improving protection against both biotic and abiotic stresses, secondary metabolites are an important source of anticancer, antioxidant, antidiabetic, immunosuppressive, antifungal, anti-inflammatory, antimalarial, anti-oomycete, antibacterial, antifever, antidiabetic, insecticidal, nematicidal, and antiviral agents [10,11,12].

The use of plants as therapies in traditional medicine is as old as mankind. Understanding how plants defend themselves is essential not only to protect our food supply, but also to take advantage of their rich chemical composition, providing numerous drugs for clinical application. Examples of those therapeutic drugs are glucoside inhibitors of sodium/potassium ATPase, which are used to treat cardiac arrhythmias and certain kinds of heart failure, and the antimalarial drugs quinine and artemisinin [13]. However, there are no plant-derived antibiotics. Actually, it is interesting to note that most of these plant-derived compounds have weak antibiotic activity—several orders of magnitudes weaker than that of common antibiotics produced by bacteria and fungi [14]. However, plants fight infections successfully. The scarcity of infective diseases in wild plants is, per se, an indication of the successful defense mechanisms they developed [9]. It becomes apparent that plants adopt a different paradigm—“synergy”—to control infections.

This review aims to provide insights on the rich variety of antimicrobial secondary metabolites (phytochemicals) from plants. Moreover, emphasis will be given to non-antimicrobial compounds, which are able to act synergistically with antimicrobials in order to promote effective defense of the plants. Their biotechnological value as potential candidates in co-therapies with antibiotics to fight multidrug-resistant (MDR) bacteria is highlighted. The main mechanisms involved in biofilm resistance to antibiotics and the use of plant molecules to control biofilms will also be reviewed.

## 2. Clinical Multidrug-Resistant Bacteria—The Beginning of the Post-Antibiotic Era

Antimicrobial agents are arguably the most successful drugs deployed in the 20th century. These drugs are indispensable in many medical treatments such as intensive care, chemotherapy, organ transplantation, care of preterm babies, and surgical procedures, which could not be performed effectively without the availability of effective antibiotics. Their use reduces human mortality and morbidity [15,16].

Today different classes of antibiotics with distinct modes of action are available to fight diverse microorganisms [17]. However, the number of resistant microorganisms, the geographic locations affected by drug resistance, and the breadth of resistance in single organisms are increasing globally. The continued evolution and spread of multiple-antibiotic resistance in human pathogens is an alarming clinical challenge. For example, around 90%–95% of *S. aureus* strains worldwide are resistant to penicillin and, in most of the Asian countries 70%–80% are also methicillin resistant [9]. Also, Gram-negative bacteria such as *Pseudomonas*, *Acinetobacter*, *Escherichia*, and *Enterobacter* spp. are rapidly becoming very problematic due to their nosocomial status and expression of MDR phenotypes, which makes the treatment of the infections difficult [18]. This rise in the frequency of resistance among human pathogenic bacteria is a complex problem driven by many interconnected factors, in particular the extensive use of antibiotics in both human and veterinary medicine, aquaculture and agriculture [19]. Even more worrying is the fact that bacteria can develop resistance to multiple classes of antibiotics simultaneously [20]. In addition, genetic resistance determinants were also detected in members of microbial communities from natural environments, raising the concern about the risk that those antibiotic resistance reservoirs might constitute to human and ecological heath [21].

Traditional antibiotics are increasingly subject to a decline in bioactivity due to the emergence of MDR bacteria, which makes it imperative to search for alternative treatments [22]. Only two classes of synthetic antibiotics were developed in the past 50 years: fluoroquinolones and oxazolidinones (linezolid). All other similar efforts to find a new antibiotic failed, which indicates that there is an extremely low probability of discovering a new drug [13].

The increase in the frequency of MDR bacteria and the subsequent absence of access to effective antimicrobial agents represents one of the most threatening health care problems with worldwide concern [15]. Understanding the mechanisms by which bacteria defend themselves against antimicrobial agents is essential to circumvent this public health threat. Many factors contribute to the emergence of resistant phenotypes to antimicrobial agents, including: the degree of the expression of resistance determinants, the capacity of the microorganisms to maintain resistance mechanisms, the capacity of transmission, the bacterial fitness, and the potential for reversibility [23].

### 2.1. Mechanisms Explaining Bacterial Resistance to Antibiotics

Antibiotic resistance can be intrinsic but can also be acquired via mutations in chromosomal genes and by horizontal gene transfer [24]. Bacterial intrinsic resistance to a particular antibiotic is the ability of a species to resist to the action of that antimicrobial agent as a result of intrinsic structural and functional characteristics [25]. Bacteria can also acquire or develop resistance to antibiotics through spontaneous mutation or by acquiring genes from other bacteria. The acquisition of exogenous genes may occur by transduction (mediated by bacteriophages); conjugation (which involve direct cell-to-cell contact and transfer of plasmids or transposon); or transformation involving the uptake of free DNA that results from bacterial lysis [15,26].

**Figure 1:**
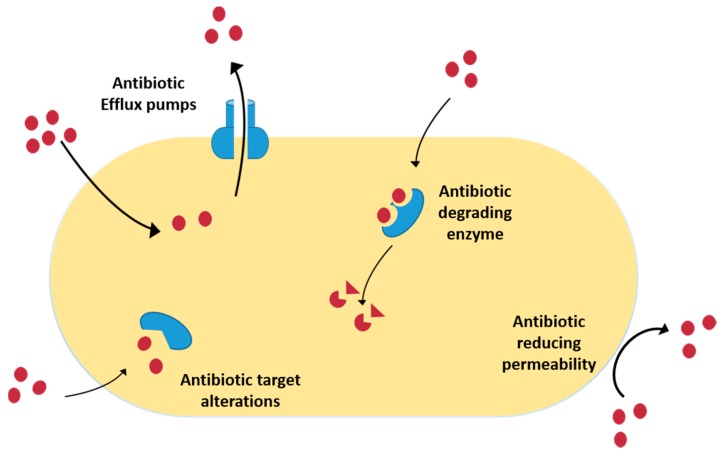
Mechanisms involved in bacterial resistance to antibiotics.

Susceptible bacteria may become resistant to antibiotics through multiple and complex mechanisms, such as prevention of access to the target by reducing permeability, increased efflux pumps (EPs) expression, degradation/modification of the antibiotics, and modification of the molecular target (Figure 1) [1]. More detailed information about all of them will be presented below.

- **Prevention of access to target by reducing permeability**—Microorganisms are surrounded by a cell envelop that constitutes a selective permeability barrier and thus can effectively offer protection from drug molecules in the extracellular environment, while providing sufficient nutrients to the cell [27]. Most of the antibiotics target intracellular processes and must be able to penetrate the bacterial membrane [28]. Reduction of the concentration of the antibiotic into the cell, due to modification of the cell surface, limits the interaction with the drug (e.g., lipid A modification) or reduces the number of entry channels (e.g., porins) [27]. In Gram-negative bacteria the outer membrane serves as a physical and functional barrier. Such an outer membrane contains an inner layer that has phospholipids and an outer layer that has lipid A. This composition reduces the permeability to many drug [25]. For bacteria belonging to the Enterobacteriaceae family, the majority of the porins are thought to function as non-specific channels; an example of this is the outer-membrane proteins OmpF and OmpC of *Escherichia coli* [15].
- **Increased efflux pumps**—Several energy-dependent systems that reduce the intracellular concentration of toxic substances were identified in bacteria. EPs are transport proteins, localized in the cytoplasmatic membrane, found in both Gram-negative and -positive bacteria as well as in eukaryotic organisms. These pump systems remove toxic compounds out of the bacterial cell in a process that does not comprise the alteration or degradation of the molecules [27]. EPs can be specific for one substrate or transport a variety of structurally dissimilar substances, such as these latter pumps associated with the occurrence of MDR bacteria [29]. Currently, the identification and characterization of EPs remains one of the major problems [30]. There are five major families of EP transporters: RND (resistance-nodulation-division); MF (major facilitator); MATE (multidrug and toxic efflux); SMR (small multidrug resistance); and ABC (ATP binding cassette). In most of the bacteria, EP genes are localized in an operon controlled by a regulatory gene [31]. In the last years, new EPs have been described, such as MdeA in *Streptococcus* *mutans*, KexD in *Klebsiella pneumoniae* and Lmrs in *Staphylococcus aureus* [32,33,34].
- **Degradation and modification of antibiotics**—Another means by which bacteria can be resistant is by destroying the active component of the antibiotics. There are three mechanisms through which bacteria inactivate the antibiotics: enzymatic hydrolysis; group transfer; or redox process [30]. Among them, antibiotic inactivation catalysed by enzymes is the main mechanism of resistance. Various antibiotics have hydrolytically susceptible chemical bonds, e.g., esters and amides, whose integrity is central to biological activity [35]. Thousands of enzymes are known to degrade and modify antibiotics of different classes, including β-lactams, aminoglycosides, phenicols and macrolides. The expansion of antibiotic classes to include derivatives that have improved properties has been reflected by the emergence of hydrolytic enzymes that have altered spectres of activity [36,37,38,39].
  The β-lactamases are broadly prevalent and clinically important resistant enzymes [40]. The β-lactamases can be classified using two systems: Ambler and Bush-Jacoby-Medeiros [24]. These enzymes are the most important mechanisms of resistance in Gram-negative bacteria and can be coded on plasmids and chromosomes [25]. The genes that codify β-lactamases can be transferred by transposons but can be found in the composition of integrons [41]. There are two distinct chemical mechanisms employed by β-lactamases to hydrolytically cleave the ring of β-lactam antibiotics: formation of a covalent enzyme intermediate followed by hydrolysis or meta-activation of nucleophilic water molecules via the Zn^2+^ centre [30,42]. Serine β-lactamase cephalosporinases are found in *Enterobacter* spp. and *Pseudomonas* spp., penicilinases in strains of *S. aureus* [24,43,44,45,46]. The enzyme metallo-β-lactamase, found in *Pseudomonas aeruginosa*, *K. pneumoniae*, *E. coli,* and on the Gram-positive bacteria *Proteus* *mirabilis* and *Enterobacter* spp., is responsible for resistance to imipenem, and the new generation of cephalosporins and penicillins [25,43]. Extended-spectrum β-lactamases (ESBLs) that are active against first-generation β-lactams can be encoded in large plasmids but can also be transferred by transposon insertion [47,48,49,50,51]. Other examples of hydrolytic enzymes include esterases, which have been linked to macrolide antibiotic resistance and fosfomycin resistance ring-opening epoxidases [25]. Hydrolytic enzymes inactivate the antibiotics before the molecule can reach their target in the bacteria. Because these enzymes require only water as a co-substrate, they can often be excreted by the bacteria [30]. The most varied family of resistance enzymes is the transferases group that inactivates aminoglycosides, chloramphenicol, streptogramin, macrolides or rifampicin. The inactivation of antimicrobial agents occurs by binding adenylyl, phosphoryl, or acetyl groups to the periphery of the molecule. Phosphoryltransferases, nucleotidyltransferases, or adenylyltransferases and acetyltransferases are aminoglycoside neutralizer enzymes [52]. These enzymes can be found in *S. aureus*, *Enterococcus faecalis* and *Streptococcus pneumoniae* [53]. Oxidation and reduction are also used by pathogenic bacteria as a mechanism of antibiotic resistance [52,54].
- **Modification of the molecular target**—The majority of the antibiotics specifically bind to their targets with high affinity, so even a small mutation in a target molecule is sufficient to influence antibiotic binding to the target [52]. Alteration of the natural antibiotic target may arise from a spontaneous chromosomal mutation resulting in single or multiple amino acid modification, or from homologous recombination with exogenous DNA containing gene segments that encode proteins with low antibiotic binding affinity [30]. In clinical pathogenic bacteria, several genes that encode for target modification of the same antibiotic were already described. One example is the methicillin resistance in *S. aureus* [24,52,55].

### 2.2. Biofilms as a Contributing Factor for the Increased Resistance to Antibacterials

The physiology and metabolic condition of the bacteria are also two important factors related to their antibiotic tolerance. Indeed, it has been observed that several antibiotics are not active against dormant cells. This phenomenon can help to also explain the resistance demonstrated by bacteria in biofilms [56]. Biofilms are common in nature and can cause numerous chronic infections, including cystic fibrosis, pneumonia, periodontitis, and varied infections associated with indwelling devices such as catheters, heart valves, orthopaedic devices and contact lenses [57,58,59,60]. In the environment and industry, biofilms are associated with biofouling and the corrosion of pipes and amplified frictional resistance to fluid movement greatly increases energy consumption. They are also associated with contamination and spoilage in food processing [61].

It is becoming increasingly clear that cells in biofilms are more resistant to antibiotics, biocides and other chemical or physical challenges compared to the same cells in planktonic state [58,62,63,64]. The development of antibiotic resistance is not well known, but recent studies have developed a variety of model systems to understand how and why biofilms are more resistant to antibiotics [63].

A great deal is known about the genetic and molecular basis of antibiotic resistance in planktonic bacteria. However, the most common mechanisms of antibiotic resistance in planktonic bacteria do not seem to be responsible for the biofilm resistance. Even susceptible bacteria that do not have a known genetic basis for resistance can be profoundly resistant when they are in a sessile state [57]. One example of this situation is *P. aeruginosa* strains that do not have the MexAB-oprM multidrug-resistance EPs, but remain resistant to ciprofloxacin in biofilms [58,65]. The rate of mutation in biofilms is significantly more frequent in comparison to planktonic cells [66]. The horizontal gene transmission is also higher in bacteria growing in biofilms [67]. These physiological characteristics explain the common occurrence of MDR bacteria in biofilms [68].

**Figure 2:**
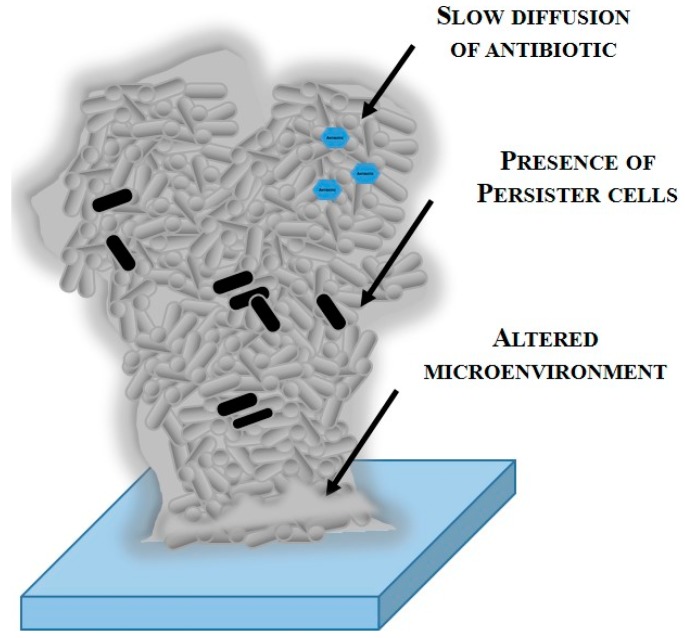
Main mechanisms of biofilm resistance to antibiotics.

Varied mechanisms have been proposed to elucidate the high resistance of biofilms including restricted antibiotic penetration, decreased growth rates and metabolism, and induction of cell biofilm–specific phenotypes known as persister cells (Figure 2) [57,58]. Biofilm resistance to antibiotics normally varies from one microorganism to another and is frequently multifactorial [69].

Biofilms can be formed by different species and genera, generally adhered to a surface and embedded within an exopolysaccharide matrix [70]. This matrix can include ions, nutrients sequestered from the environment and extracellular enzymes (β-lactamases, proteases polysaccharases). Furthermore, it acts as a diffusion barrier and reaction sink (neutralizer) minimizing the intracellular concentration of the antibiotic as a result of poor penetration into the biofilm [63,69]. Indeed, one simple mechanism of biofilm protection is the retention of the antibiotic in the biofilm fluid bathing [71]. Suci et al. [72] observed a delayed penetration of the fluoroquinolone ciprofloxacin into *P. aeruginosa* biofilms. Hoyle and collaborators [73] demonstrated that bacteria in biofilms are 15 times more resistant to tobramycin than the planktonic cells. However, the decrease of the antibiotic mobility in biofilms was insufficient to explain the level of resistance in aggregated bacteria [57].

Another factor explaining the high resistance of biofilms is the altered chemical microenvironment within the biofilm. The decrease of oxygen and the nutrient gradient that exist inside of biofilms, between the surface and the deeper layers, can cause altered metabolic activity and lead to slow/stationary growth [69,74,75]. The cytotoxic action of the majority of the antimicrobial agents is growth-dependent. For example, β-lactam antibiotics are only active against actively growing bacteria [57,63]. Even single biofilm species are metabolically heterogeneous [60]. Gilbert and colleagues studied growth rate-related effects under controlled growth conditions for biofilm and planktonic cells of *P. aeruginosa*, *E. coli* and *Staphylococcus epidermidis*, and concluded that the decrease of the cell growth rate increased the resistance to tobramycin and ciprofloxacin [76,77,78]. Additionally, the decrease of the growth rate was associated with resistance to cetrimide in *E. coli*, ciprofloxacin in *S. epidermidis*, and tobramycin and penicillin in *P. aeruginosa* [69].

A reduced percentage of biofilm cells remain active after prolonged exposure to a high concentration of antibiotics [79]. These bacterial phenotypes are called persister cells that show a reduced growth rate and high resistance to antimicrobial agents [80]. The formation of these cells can highly contribute to an increased antibiotic resistance in biofilms [79]. Persister cells are not exclusive to biofilms. However, the frequency of occurrence is much higher within sessile communities than among planktonic communities [57].

### 2.3. Drug-Resistant Microorganisms—from Environment to Clinic

Before dealing with one problem, it is first necessary to understand it. Contrary to the previous opinion that antibiotic-resistant bacteria are a problem restricted to clinical settings, it has been recently recognized that antibiotic resistance and the occurrence of genetic determinants are ubiquitous and occur naturally in several ecosystems, from terrestrial to aquatic. Indeed, some studies showed that these genetic elements are even present in soils from the pre-antibiotic era (preserved in a frozen state) or in environments that never contacted antimicrobials. Basically, they can be found in all sites that have contacted or are in contact with microorganisms [81]. Therefore, the dissemination of antibiotic resistance genes is almost inevitable and non-clinical environments have been highlighted as an important factor that contributes to this occurrence [82].

Our current arsenal of antibiotics is most exclusively from microbial sources. If we are indeed taking advantage of the natural weapons that microorganisms have been producing to fight with each other for millions of years, perhaps one could expect that by co-evolution, resistance to these compounds would also appear naturally. Indeed, antibiotic-resistant genes have been characterized in coliforms from glacial water and ice in the Arctic, which are estimated to be more than 2000 years old [83]. The question then is, does the environmental resistome intersect with the resistome of clinical pathogens or are they distinct? Wright [84] extensively reviewed connections that show a clear link between resistance in the environment and the clinic: the CTX-M extended-spectrum β-lactamase appears to come from chromosomal genes of the environmental genus *Kluyvera;* the *qnrA* gene associated with fluoroquinolone resistance has an environmental reservoir in the aquatic bacterium *Shewanella algae;* the gene cluster that confers resistance to vancomycin (a glycopeptide antibiotic) in *Enterococci* and *Staphylococci* has been identified in environmental *Bacilli*, in glycopeptide-producing and non-producing environmental bacterial strains [84]. Additionally, culturable bacteria in soil were found to encode enzymes that degrade or inactivate antibiotics [85]. Dantas and colleagues [21] isolated hundreds of soil bacteria with intrinsic multidrug-resistant phenotypes with the capacity to grow on antibiotics as a sole carbon source. In one soil species, over 400 actinomycetes cultured from forest, agricultural and urban soils were found to have varied resistance profiles and some of them even exhibited resistotypes that had not been seen before [86]. Forsberg et al. [87] describes multidrug-resistant soil bacteria containing resistance cassettes against five classes of antibiotics (β-lactams, aminoglycosides, amphenicols, sulfonamides, and tetracyclines) that have a perfect nucleotide identical to genes from diverse human pathogens. The Handelsman group identified and characterized several new antibiotic resistance genes from soil bacteria including aminoglycoside acetyltransferases, tetracycline efflux [88], β-lactamases [89], fenicol efflux proteins [90], etc. Even in the subsurface, bacteria resistant to antibiotics (nalidixic acid, mupirocin or ampicillin) were found to be common by Brown et al. [91].

Despite microorganisms continuing to be the major source of therapeutic antibiotics, the possible continuing spread of new resistance mechanisms to new antibiotics is enormous. Therefore, new antimicrobial strategies and new sources of active compounds are urgently needed.

## 3. Non-Conventional Strategies to Treat Drug-Resistant Bacteria

Several non-conventional ways of treating infections have shown promising results in preclinical studies and a few of them have succeeded in demonstrating significant clinical outcomes [92]. The β-lactam antibiotics are the largest antibiotic family used in medicine. However, their use is readily compromised partly because of the production of β-lactamases, such as TEM-1, TEM-2, or SHV-1 [93,94]. Many strategies for avoiding, inhibiting, or bypassing resistance mechanisms in pathogens have been attempted [95]. Molecules that block antibiotic resistance can have significant clinical impact and therefore rescue antibiotic activity. Therefore, they are called antibiotic adjuvants. The most notable successes in this strategy have been with the β-lactam antibiotics. Clavulanic acid, sulbactam, tazobactam, and avibactam are β -lactamase inhibitors that have little intrinsic antibacterial activity but inhibit the activity of a number of plasmid-mediated β-lactamases [96]. Interestingly, clavulanic acid is produced by *Streptomyces clavuligerus*, which also produces several β-lactam antibiotics. However, organisms have found a way to outsmart us by producing ESBLs, AmpC β-lactamases, and carbapenemases, demonstrating a critical need for new antibiotics [96].

To date, research to extend this approach to other classes of antibiotics has not been successful. To explore new compounds able to inhibit cephalosporinase and other enzymatic activities, a possibility is to facilitate the diffusion of antibiotics through the bacterial envelope in order to increase their intracellular concentration. Some of them, e.g., polycationic cyclic lipopeptides and cationic antimicrobial peptides, have been assayed in combination with common antibiotics to combat resistant clinical strains [97]. Microbial EPs have become broadly recognized as major components in mediating multidrug resistance in clinical isolates from varied geographic locations and populations. Some EPs may be selective for one substrate, such as tetracycline. However, EPs are generally promiscuous, and a variety of low-molecular-weight compounds with limited structural similarities may be substrates for the same pump [98]. The overexpression of these pumps and synergy with other drug resistance mechanisms hampers effective antimicrobial treatment [99]. Indeed, the multi-specificity of efflux transporters can drive the acquisition of additional mechanisms of antibiotic resistance such as mutation of antibiotic targets or secretion of enzymes that degrade antibiotics (e.g., β-lactamases) [98]. One plausible antimicrobial therapy could be the combination of antibiotics with small molecules that block multidrug efflux systems known as EP inhibitors (EPIs) [22]. Such a combination could restore the activity of standard antibiotics. EPIs would allow the enhancement of antibiotic uptake to overcome drug efflux [100], and restore the antibacterial treatment by decreasing the intrinsic bacterial resistance to antibiotics, reversing the acquired resistance associated with EP overexpression, and reducing the frequency of the emergence of resistant mutant strains [99,101]. There are several ways of targeting EPs: the alteration of pump gene expression, the inhibition of the membrane assembly of the pump component, blocking outer membrane exit duct, and collapsing the energy-driven source, among others [97]. In recent years, a large number of MDR inhibitors have been discovered and patented but the process of discovery, testing and commercialization is rather slow [99]. The compounds categorized as EPIs include: antibiotics and derivatives; antipsychotics; antihistaminics; antihypertensives; plant extracts and constituents; and peptides produced by insects [101,102,103,104,105]. Despite many of them showing in vivo activity, so far only one reached human clinical trials (phase II), because there are still some problems that preclude their clinical use. Also, another one of the main reasons is related to the lack of information on the mode of action of the majority of EPIs [101,103,104,105]. Globomycin from *Streptomyces hagronensis* blocks the functional assembly of some components of EP such as AcrA; carbonyl cyanide *m*-chlorophenylhydrazone and potassium cyanide seriously affect the energy level of the bacterial membrane; and Phe-Arg-β-naphthylamine effectively inhibits the efflux mechanism associated with quinolones, among other efflux systems [98].

Other possibilities of non-conventional antibiotic treatments include the use of compounds that inhibit quorum sensing (QS), target pathogen virulence, or prevent biofilm formation or the adherence to the host tissues, etc. [92].

## 4. The Use of Plants as Sources of Antibiotic Adjuvants

During the last few years, the increasing incidence of drug-resistant pathogens has drawn the attention of the pharmaceutical and scientific communities towards studies on the potential antimicrobial activity of plant-derived substances. This allowed a resurgence in the use of herbal medicines worldwide [94]. Plant-based systems continue to play an essential role in healthcare, and their use in different cultures has been extensively documented [106]. For example, goldenseal (*Hydrastis canadensis* L.) is used to treat inflammation and infection. Its antibacterial activity in vitro has been attributed to alkaloids, the most abundant of which is berberine [107].

Plants represent a vast source of interesting compounds. The world of higher plants, especially rainforests, has only been partly explored for the isolation of new compounds and it is estimated that only ~6% of the approximately 300,000 species of higher plants have been pharmacologically investigated, and only ~15% phytochemically [106]. A potential source of bioactive compounds is also the reinvestigation of metabolites previously assumed to be inactive, which represent ~60% of the known metabolites [108]. For example, naftifine, an Food and Drug Administration (FDA)-approved antifungal drug, was found to be a lead compound for potent CrtN inhibitors and to attenuate the virulence of a variety of clinical *S. aureus* isolates, including methicillin-resistant *S. aureus* (MRSA) strains, in mouse infection models [109]. The total number of marketed drugs used in human therapy is estimated to be ~3500 compounds, representing less than 0.01% of all known chemical compounds [108].

Synergistic interactions are of vital importance in phytomedicine to explain the efficacy of apparently low doses of active constituents in an herbal product [110]. This concept is based in the idea that an extract of a plant offers advantages over a single isolated ingredient. Synergistic effects can be produced if the interaction between constituents of the extract results in increased solubility and, thereby, enhances the bioavailability of active compounds. Interaction with different targets by the different defensive components of the extract also promotes synergistic effects and enhances the defensive system of plants. For example, in tomato, alkaloids, phenolics, proteinase inhibitors, and the oxidative enzymes act synergistically, affecting insects during ingestion, digestion and metabolism; in *Nicotiana attenuata*, trypsin proteinase inhibitors and nicotine expression contributed synergistically to the defensive response against *Spodoptera exigua* (Hub.) [5]. Most compounds that are synthesized in response to pathogen invasion are not even necessarily antimicrobial. Such compounds might have a regulatory function, indirectly increasing the level of resistance of the plant [14]. A special synergy effect can then occur when antibiotics are combined with an agent that antagonizes bacterial resistance mechanisms [111]. The utility of plant products as antibiotic potentiators and virulence attenuators has been intensively studied [8].

Plants have been extensively reported to be able to produce drug-resistance inhibitors in order to ensure the delivery of the antimicrobial compounds. Since resistance mechanisms are greatly shared between environmental and clinic settings, as described previously, these interesting compounds may have direct applications on clinical infections. There have been other efforts to uncover synergistic interactions between antimicrobial and non-antimicrobial molecules, much of it in the antifungal field. Some examples include marked synergy between azole antifungals and a group of small molecules called citridones, produced by *Penicillium* sp. [100]. The activity of rhein, the principal antimicrobial from rhubarb, was potentiated 100- to 2000-fold (depending on the bacterial species) by disabling MDRs [14]. Comparable antimicrobial potentiation was observed with plumbagin, resveratrol, gossypol, coumestrol, and berberine [14]. The ability of crude extracts of plants and phytochemicals to potentiate the activity of antibiotics has been observed, reported and reviewed by some researchers [9,100,110,112,113,114]. Few efforts were carried out in this direction which led to the identification of some interesting compounds present in different medicinal plants such as reserpine, protocatechuic acid, gallic acid, ellagic acid, carnosic acid, totarol, rutin, quercetin, morin, biochanin A, genistein, and myricetin, berberine, etc. [112,115,116,117]. The ability to pump antibiotics out of cells is a common feature of most environmental microbes and their pathogenic relatives. Therefore, EPIs may be easily found in nature. Bioassay-guided isolation and structural determination of compounds from plant sources have yielded a number of EPIs [22]. It has been established that disabling EPs leads to a striking increase in the activity of a wide array of plant secondary metabolites. For example, *Berberis* medicinal plants that produce the plant antimicrobial berberine also synthesized an inhibitor of the *S. aureus* NorA EP identified as 5′-methoxyhydnocarpin [22]. Stavri et al. [118] described different bacterial EPIs, such as the plant alkaloid reserpine, berberine and methoxylated flavones and isoflavones, which revealed putative interfering activity on efflux. In *Rhizobium etli*, an operon that appeared to code for an RmrAB MDR, is activated by a number of plant phytoalexins; in *Agrobacterium tumefaciens*, coumestrol, an antifungal phytoalexin of soybeans, was found to induce the expression of an LfeAB MDR, and a mutation in the pump increased the level of accumulation of coumestrol in the pathogen [14]. The compound 4′,5′-*O*-dicaffeoylquinic acid from *Artemisia absinthium* was identified as an EPI with the potential of targeting efflux systems in a wide panel of Gram-positive human pathogens [22].

The vast majority of EPIs identified so far are active against Gram-positive bacteria, particularly *S. aureus*. There is a dire need to search for EPIs that are effective in rendering MDR Gram-negative bacteria [18]. The very few EPIs that are active against Gram-negative bacteria may be cytotoxic. Recent data indicate that the AcrAB-TolC (in Enterobacteriaceae) and MexAB-OprM (in *P. aeruginosa*) EPs are involved in the resistance of Gram-negative bacteria to most of the natural products [119]. It was hypothesised that herbal medicinal products may contain EPIs for Gram-negative bacteria, since most plant bacterial pathogens are Gram-negative. Garvey et al. [120] extracted *Levisticum officinale* and identified falcarindiol, oleic acid and linoleic acid in the fractions displaying the greatest synergy with five antibiotics; possibly by an efflux inhibition of AcrAB-TolC. The essential oil of *Helichrysum italicum* significantly reduces the multidrug resistance of *Enterobacter aerogenes*, *E. coli*, *P. aeruginosa*, and *Acinetobacter baumannii* [18]. Geraniol, found in the essential oil, significantly increased the efficacy of β-lactams, quinolones, and chloramphenicol [18].

Parallel studies on crude plant extracts and their synergistic effect with conventional antibiotics against MDR pathogens can also be widely found. Darwish et al. [121] demonstrated that the efficacy of gentamicin and chloramphenicol against *S. aureus* was reportedly improved by the use of some Jordanian plant materials. Ahmad et al. [122] reported that crude extracts of Indian medicinal plants were synergistic with tetracycline and ciprofloxacin against ESBL-producing MDR-enteric bacteria. Touani et al. [123] found that the methanol extracts of *Brassica oleacera* var. *butyris*, *Brassica oleacera* var. *Italica*, *Capsicum frutescens* var. *facilulatum* and *Basilicum polystachyon* showed synergistic effects (fractional inhibitory concentration  ≤0.5) with an average of 75.3% of the tested antibiotics against MDR Gram-negative bacteria [123]. Barreto et al. [124] showed that the ethanol and hexane extracts of the stem bark of *Anadenanthera colubrine* (Vell.) Brenan var. *cebil* enhanced the activity of neomycin and amikacin against *S. aureus* SA10. Barreto et al. [125] also showed a minimum inhibitory concentration (MIC) reduction (by 10-fold) of neomycin and amikacin when combined with the essential oil from *Lippia origanoides* H.B.K. against MRSA. Betoni and coworkers [114] also observed synergistic interactions between extracts of Brazilian medicinal plants and eight antibiotics on *S. aureus*. Many examples of synergistic activities in which essential oils have been found to reduce the minimum effective dose of antibiotics in the treatment of infections are described by Yap et al. [94]. Aumeeruddy-Elalfi et al. [126] reported the synergistic effect of the essential oils of *Pimenta dioica*, *Psidia arguta* and *Piper betle* when combined with gentamicin against * E. coli* and *S. epidermis*. Fankam et al. [127] investigated the antibacterial and antibiotic-resistance-modifying activities of the methanol extracts from *Allanblackia gabonensis, Gladiolus quartinianus* and *Combretum molle* against 29 Gram-negative bacteria including MDR phenotypes. Percentages of antibiotic-modulating effects ranging from 67% to 100% were observed against MDR bacteria when combining the leaves extract from *C. molle* (at one-half MIC and one-fourth MIC) with chloramphenicol, kanamycin, streptomycin and tetracycline [127]. Tankeo et al. [128] found synergistic effects against a panel of Gram-negative bacteria, including MDR phenotypes expressing active EPs, obtained with *Beilschmedia acuta* bark extract and tetracycline as well as with the extract of *Polyscias fulva* leaves (at one-half MIC) and tetracycline and kanamycin. Also, in the extracts of wild mushrooms, especially from *Mycena rosea* and *Fistulina hepatica*, interesting synergistic activities between these extracts and commercial antibiotics (penicillin, ampicillin, amoxicillin/clavulanic acid, cefoxitin, ciprofloxacin, cotrimoxazol, levofloxacin) against *E. coli*, ESBL *E. coli* and MRSA were observed [129].

## 5. Non-Conventional Strategies to Treat Bacteria Growing within a Biofilm

**Figure 3:**
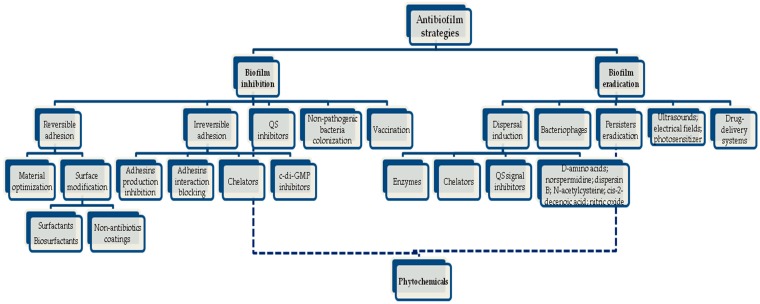
Main antibiofilm strategies. The suggested approaches can be divided in two major lines of action: some are meant to prevent biofilm formation and others to eradicate established biofilms, and they can comprise the application of physical, chemical and biological methods. Some of them such as QSI, chelating agents and the use of natural compounds from plants (phytochemicals) can be applied for both biofilm inhibition and eradication. Although the second messenger c-di-GMP is mainly involved in the transition of the planktonic to sessile state, it can be also used as a target to disperse biofilms. The use of photodynamic therapy and nanoparticles as drug carriers is not specific for biofilm removal, as they can be useful to inhibit biofilm formation.

The concern in developing antimicrobials with improved efficacy against pathogenic bacteria that are highly resistant to antibiotics and host immunity has been mainly directed at planktonic bacteria without taking into account the specificities of the sessile lifestyle [130]. However, there is no doubt that new sources of antimicrobials and strategies promoting effective biofilm inhibition and/or eradication are crucial. Through the years, several strategies have been proposed, but they have not been sufficiently powerful and additional therapeutic solutions continue to be needed [131]. During the search for novel drugs and alternative approaches for the treatment of biofilm-related infections, it is helpful to identify factors that impair biofilm formation or disturb its structure, based on both physical and biological characteristics. In this way, targeting the different stages of biofilm development (e.g., adhesion, motility, production of extracellular polymeric substances (EPS) and QS phenomena) and inducing biofilm inactivation and removal by means of weakening, dispersion or disruption can be a promising strategy [130,132,133]. Current investigations are increasingly focusing on the discovery of compounds and techniques that are able to change the phenotype of the bacteria without inducing modification at the genetic level, thus avoiding selective pressure, which otherwise could lead to the development of resistance [134]. Diverse novel strategies to deal with unwanted biofilms have already been identified [135,136,137,138,139,140] (Figure 3).

To impede the establishment and development of sessile bacterial communities there are several “prophylactic” methods, including [131,135,136]:

(a) Inhibition of bacterial reversible adhesion by optimization of the physicochemical properties of the materials used in medical devices and implants, and surface modification using surfactants/biosurfactants and other types of non-antibiotic coatings;
(b) Inhibition of irreversible adhesion by interference with the production of adhesins, blocking of the adhesins’ interaction with their receptors, use of chelating agents that inhibit the transport of essential metals to the interior of cells, thus stopping biochemical pathways that are crucial for biofilm formation, and inhibition of nucleotide signalling biosynthesis such as cyclic diguanosine monophosphate (c-di-GMP), which can maintain bacteria in the planktonic state;
(c) Interference with the bacterial communication through the use of QS inhibitors (QSI); the use of non-pathogenic bacteria which can compete with pathogens by producing toxins (e.g., bacteriocins) or other substances, thus preventing colonization; and vaccination in order to produce antibodies against antigens of bacterial biofilms, preventing the evolution of infection.

On the other hand, if biofilm formation cannot be prevented there are methods for biofilm disassembly [131,135,137,138]:

(a) Induction of dispersal by the application of enzymes (e.g., DNAse I, proteinase K, tripsin, lysostaphin, amylase, lyase and lactonase), divalent metal chelators, QS signal inhibitors, and other molecules such as d-amino acids (e.g., d-leucine, d-methionine, d-tyrosine and d-tryptophan), norspermidine, dispersin B, *N*-acetylcysteine, cis-2-decenoic acid, and nitric oxide;
(b) The use of bacteriophages;
(c) Eradication of persister cells (e.g., combined application of sugars or silver with antibiotics and/or an increase in the production of reactive oxygen species).

Other promising antibiofilm approaches have also been suggested and consist in the application of ultrasounds, electrical fields and photosensitizers to enhance the activity and transport of the antibiotics through biofilms. In addition, nanoparticles, polymer-based antimicrobials, liposomes and aerosols can be also used as drug-delivery systems, permitting the release of the drugs in a controlled manner [137,139,140].

Last but not least, natural compounds from plants have drawn the attention of the scientific community, as they are considered a green and sustainable source of new molecules that have shown to be effective biofilm inhibitors. The structural diversity displayed by phytochemicals associated with their multi-target mode of action is also of interest and differs significantly from the properties of conventional antibiotics. These distinctive properties can help to overcome the resistance problem. In fact, there is no evidence on the occurrence of bacterial resistance to phytochemicals [56].

## 6. Plant-Based Strategies to Deal with Unwanted Biofilms

### 6.1. Phytochemicals as Quorum-Sensing Inhibitors

Novel strategies should ideally target cellular processes responsible for pathogenesis and virulence, recognized as “antipathogenic” or “antivirulence” therapies, instead of targeting bacterial growth [141]. Indeed, the use of anti-infective drugs could have the advantage of reducing bacterial adaptability to the host environment, facilitating the host immune system to combat the infection and reducing the strong selective pressure exerted by conventional antibiotics [142].

In many bacteria, pathogenicity and/or virulence is controlled and coordinated by a process of intercellular communication named QS. Several Gram-positive and Gram-negative bacteria use QS to coordinate the expression of group-beneficial phenotypes and regulate physiological activities, which promote pathogenesis and enable bacteria to resist to antimicrobial compounds. They include, among others, the production of virulence factors, secondary metabolites and the formation of structured microbial communities such as biofilms [143,144]. Indeed, QS systems are integrated into some processes important to biofilm formation and differentiation [56]. Thus, QSI are also a possible key to overtake the limitations of in-use antibiotics to treat biofilm infections [145,146]. The proposed advantages for the infection control by QS inhibition are based on the fact that QSI do not suppress the growth of cells and hence will not exert selective pressure to develop resistance. In this sense, among the many virulence traits that can be the possible targets of the anti-infective agents, QS circuits seem to be the most attractive [147].

All QS systems share a mechanism comprising signal production, accumulation and detection. Whatever the efforts employed to disrupt this phenomenon, all strategies will be based in inhibiting one of these steps [141,148]. QS inhibition works equally in planktonic and sessile bacteria [130]. In sessile bacteria, QS is a key component of communication that is linked with diverse events. The interruption of QS signaling pathways can prevent initial biofilm formation and alter its progression through the inhibition of the secretion of adhesins or cellular appendages, which affect bacterial motility, adhesion to surfaces, cell auto and coagregation, formation of microcolonies, and inhibition of the EPS production [56]. Some QSI and QS signals can also be used to induce biofilm dispersal [135]. Indeed, it has been documented that signaling molecules mediate several aspects of the biofilm dynamic (e.g., heterogeneity, architecture, stress resistance, maintenance and sloughing) [149]. Their blockage can increase biofilm susceptibility to antibiotics and host defences and thus favours the use of low doses of antibiotics, leading to an easier eradication [68,150,151]. Besides, they avoid the indiscriminate use of broad-spectrum antibiotics that are frequently more toxic and expensive [146,152].

Despite the great potential that QS inhibition has revealed for the control of infections, our knowledge about QS systems and QSI are still limited. Moreover, the majority of QSI identified so far are still at the preclinical stage and have not been thoroughly explored with respect to their cytotoxicity [152]. Thus, despite the massive efforts made to date in the field of QSI research, clinical applications remain far away. The current quest is therefore aimed at discovering new QSI with reduced side effects in human health. In this sense, the main criteria used for the selection of an effective QSI are based on its specificity for a given QS regulator with few or no adverse effects on the bacteria or host [148].

Several screenings have shown that natural products, particularly phytochemicals, are an interesting source of QSI [153]. They have been recognized as a large and attractive repository of QSI, offering a vast chemical diversity with structural complexity and biological activity [56,154]. In fact, they resemble what is considered an “ideal” QSI, which includes being chemically stable, highly effective, low-molecular-mass molecules, and being harmless to health [148,155]. Therefore, phytochemicals with QS inhibition activity can be promising tools to help the treatment of bacterial infections, including those that are biofilm-related, in an era where the availability of effective antibiotics is no longer guaranteed. Table 1 shows examples of recent studies on the use of crude extracts and/or isolated compounds from plants as biofilm modulators by targeting bacterial signaling pathways.

The identification of QSI among natural foods such as fruits and edible plants is of particular interest, as they constitute an integral part of human and animal diets and normally are nontoxic and readily available [156,219]. Good examples are the extracts from *Allium sativum*, commonly known as garlic. In vitro studies accomplished with these extracts demonstrated greater susceptibility of *P. aeruginosa* biofilms to treatment with the antibiotic tobramycin [220]. It was also observed that the intraperitoneal injection of garlic extracts enabled the clearance of pulmonary *P. aeruginosa* biofilm infections in a mouse model [221]. The aforementioned properties attributed to garlic are apparently due to QS inhibition and were initially associated with the presence of allicin and related derivatives [143,222,223], and later with ajoene [143,197]. Additionally, ginger (*Zingiber officinale* Roscoe) extracts and their main bioactive phenolic derivatives ([6]-gingerol, [6]-shogaol and zingerone) are a promising source of QSI [205]. Ginseng in general and particularly the species *Panax ginseng* also provide components (e.g., saponins, ginsenosides, and polysaccharides) with QS inhibitory properties and with the ability to disrupt biofilms [193,194]. Furthermore, extracts from pineapple, plantain and sapodilla revealed quorum-quenching (QQ) effects, and thus they had similar effects in the production of virulence factors and biofilm formation, without affecting the bacterial growth [156].

QSI were also identified in a wide variety of medicinal plants worldwide. For instance, *Centella asiatica* L. is traditionally used as a medicinal herb for the treatment of skin problems in Ayurvedic, African and Chinese medicine, due to its recognized pharmacological properties. In order to investigate its QSI potential, Vasavi et al. [171] conducted a study with reporter strains of *Chromobacterium violaceum* and *P. aeruginosa*. They showed that the ethyl acetate fraction from ethanol extracts completely inhibited violacein production in *C. violaceum* with reduced effect on its growth. Additionally, this fraction inhibited several QS-regulated phenotypes in *P. aeruginosa* (e.g., pyocyanin production, proteolytic and elastolytic activities, swarming motility and biofilm formation). In the same way, these authors studied the QS inhibitory potential of other medicinal plants (*Psidium guajava* L., *Adenanthera pavonina* L., *Syzygium cumini* L., and *P. dioica* L.) and also found positive results in terms of QSI against both microorganisms [169,170,172]. According to the studies of Koh et al. [157], other traditional Chinese herbs, namely *Prunus armeniaca, Prunella vulgaris, Nelumbo nucifera, Panax notoginseng*, *Punica granatum,* *Areca catechu*, and *Imperata cylindrical*, can be an interesting source of new QSI. Furthermore, extracts from *Amphipterygium adstringensi*, an endemic tree commonly used in traditional Mexican medicine, demonstrated potential to inhibit QS and to decrease the expression of QS-controlled virulence factors in the two model bacteria referred to above [165]. Extracts from Korean indigenous plants also exhibited QSI against *C. violaceum* and *P. aeruginosa* bioreporters [166]. Recently, Shukla and coworkers [186] found that tannin-rich crude extracts from six Indian medicinal plants (*Phyllanthus emblica*, *Terminalia bellirica*, *Terminalia chebula*, *P. granatum*, *Syzygium cumini*, and *Mangifera indica*) commonly used in Ayurveda showed QQ properties against *C. violaceum* and *S. aureus* at sub-inhibitory concentrations. Anti-QS activity was also obtained against *S. aureus* with methanolic extracts rich in pentacyclic triterpenes (ursene and oleanene derivatives) from the Italian medicinal plant *Castanea sativa*. These extracts inhibited the haemolytic activity and the production of δ-toxin by *S. aureus*. Although at a lesser extent, *S. aureus* biofilm formation was also repressed [181]. Moreover, other extracts from *Cymbopogan citrates* [162], wheat bran [175] and *Liriodendron hybrid* barks [182] with QS inhibitory activity inhibited biofilm establishment and/or eradicated pre-established biofilms of *S. aureus*, including those of MRSA clinical isolates. Extracts from *Salvadora persica* revealed promising QQ effects against cariogenic isolates of *S. mutans* through the interference of QS regulators (OmpP and Lux proteins). As a consequence, the initial cell attachment and biofilm formation were affected [160]. The crude extract and ethanolic fraction from *Emblica officinalis* also demonstrated QS inhibition potential against *S. mutans*, inhibiting cell adhesion and biofilm formation as well. Furthermore, it was also observed that this extract/fraction suppressed the expression of genes related to biofilm formation [161]. Interesting QS inhibition outcomes were achieved with the crude extract and methanolic fraction from Z*. officinale* [184]. Interaction with the OmpR QS regulator and thus the downregulation of the glycosyltransferase expression and *S. mutans* biofilm formation inhibition were obtained with methanolic extracts from *Achyranthes aspera* [164]. Moreover, a rich polyphenol extract obtained from *Usnea longissimi* demonstrated the ability to reduce the secretion of several QS-regulated virulence factors (acid production, ATPase, enolase, lactate dehydrogenase, protease, total exopolysaccharide content and glucosidase) and biofilm formation by *S. mutans* [183]. In a study conducted by Bhargava et al. [185], the production of QS-mediated virulence factors in *A. baumannii* was compromised after exposure to *Glycyrrhiza glabra* extract. *Euodia ruticarpa* extract, where the main components are evodiamine, rutaecarpine and evocarpine, displayed QS inhibitory activity against *C. jejuni* by the disruption of AI-2 production and thus cell adhesion/biofilm formation [189].

**Figure 4:**
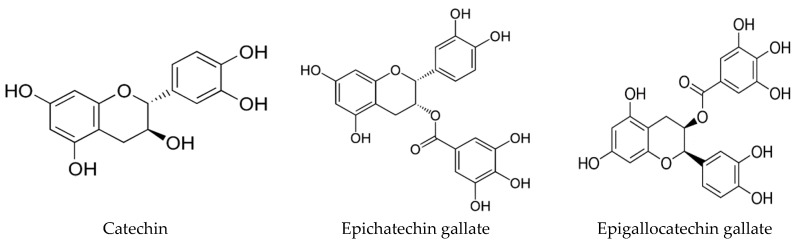

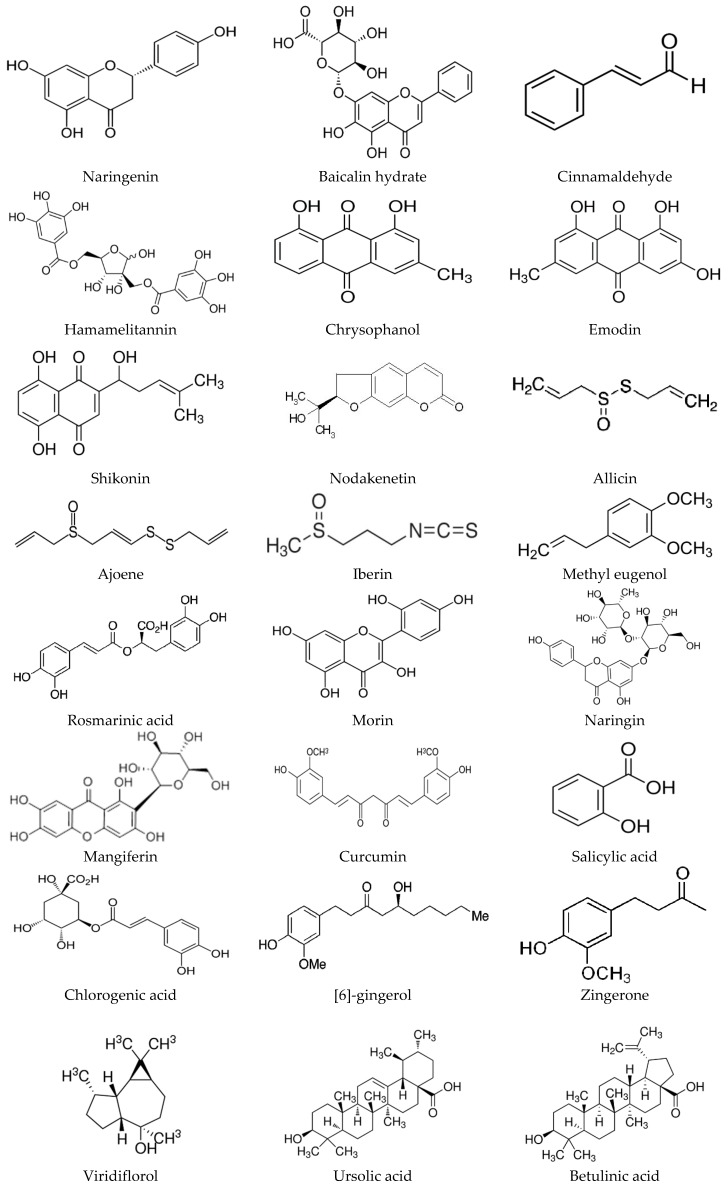

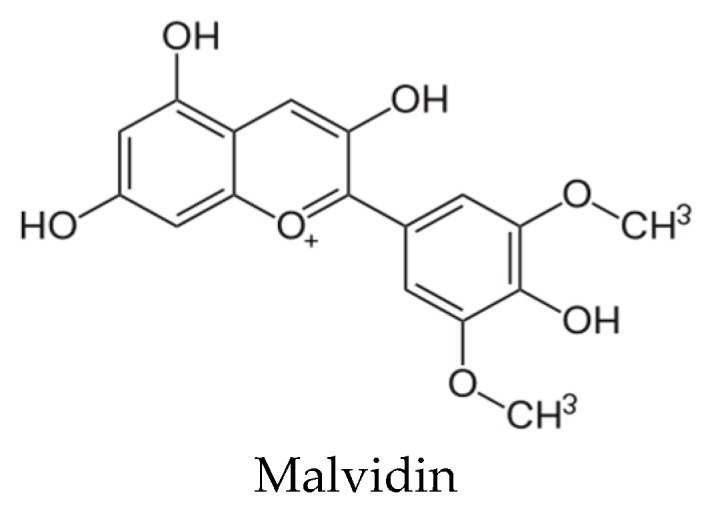
Examples of plant-based molecules able to inhibit QS-regulated processes.

Most of the studies described above concerning medicinal plants were performed with crude extracts, whose specific compounds responsible for QS inhibitory activity are almost always unknown. Subsequently, some examples of works in which pure compounds were identified and/or tested will be described. Moreover, the structures of some phytochemicals with QS inhibitory properties are also provided (Figure 4). Based on in silico screening, QQ compounds were found in Chinese medicinal herbs that had already demonstrated the potential to inhibit biofilm formation [196]. Among all of the compounds identified, emodin displayed the most interesting results. In addition to its ability to inhibit biofilm formation, emodin induced proteolysis of the *E. coli* QS signal receptor TraR and increased the activity of ampicillin against *P. aeruginosa*. A study conducted by Zhang et al. [173] exploited the anti-biofilm and QSI of *Rosa rugosa* tea polyphenol extract, where the main components are polyphenols and flavonoids. This extract demonstrated the potential to inhibit QS-controlled violacein production in *C. violaceum* without significantly affecting its growth. Moreover, the inhibition of the swarming motility and biofilm formation in *E. coli* and *P. aeruginosa* was also achieved. Brango-Vanegas et al. [202] screened extracts from leaves of *Cecropia pachystachya* Trécul for QS inhibition and found that these extracts and their glycosylflavonoids (chlorogenic acid, isoorientin, orientin, isovitexin, vitexin and rutin) showed QSI activity against *C. violaceum* and *E. coli*. Other examples of plant QSI are sesquiterpenoid viridiflorol and triterpenoids ursolic and betulinic acids, obtained from the liverwort *Lepidozia chordulifera*. These compounds also exhibited the potential to inhibit the biofilm formation of both *P. aeruginosa* and *S. aureus* and to reduce the elastolytic activity in *P. aeruginosa* [209]. In a work developed by Brackman and coworkers [195], baicalin hydrate, cinnamaldehyde and hamamelitannin demonstrated the ability to improve the susceptibility of biofilms from *P. aeruginosa*, *B. cenocepacia* and *S. aureus* (including MRSA) to treatment with conventional antibiotics (tobramycin, clindamycin and vancomycin). Furthermore, these compounds enhanced the survival of infected *C. elegans* and *G. mellonella*. More recently, Brackman et al. [211] also observed increased in vitro and in vivo susceptibility of MRSA biofilms to the antibiotic vancomycin with QSI hamamelitannin. This effect can be related to its potential to interact with the TraP receptor, affecting the release of extracellular DNA, an event of importance for biofilm formation. Potentiation of the *K. pneumoniae* biofilm susceptibility to conventional antibiotics was attained with malvidin obtained from a methanolic extract of *Syzygium cumini*. Furthermore, the inhibition of EPS synthesis and biofilm formation was obtained [210].

### 6.2. Phytochemicals as Biofilm Metal Chelators

Metal ions (e.g., calcium, magnesium, copper, manganese, zinc and iron) are involved in several biological processes crucial for the growth and survival of the microorganisms in their own environment. Furthermore, they are strictly correlated with the ability of the bacteria to invade or colonize host tissues, causing disease. During infection, bacteria employ a variety of systems to ensure metal uptake and availability, according to their physiological needs. In this sense, hosts use some defense mechanisms in order to remove these nutrients and thus limit their access to the invading pathogens. On the other hand, infected hosts can also induce toxicity in bacteria by overloading them with high doses of metals, promoting their death. Consequently, refined detoxification systems such as EPs have been developed and activated in bacteria to eliminate metals in excess [224].

In addition to the key role that metals in general and iron in particular play in pathogenesis and virulence [224], they have also been associated with biofilm formation, as they serve as signaling factors [225]. This is supported by previous studies where it was suggested that iron sensing is mainly involved in the first steps of biofilm formation (cell attachment and microcolony formation), as they are vital for bacterial cell growth and adherence [226,227]. Further, some experiments showed the importance of iron to the release of DNA, one of the major biofilm matrix components in *P. aeruginosa*, which in turn is QS controlled [228]. It has been also described that iron is required for *P. aeruginosa* biofilm formation, because when it is provided at reduced concentrations, the obtained biofilm is less structured and consists of thin layers of cells. The observed impairment in the biofilm formation could be reversed by the addition of iron, emphasizing its importance in the maturation of these sessile communities [225,229]. However, this cannot be generalized and extrapolated for other bacteria. Indeed, in a study performed by Johnson et al. [230], *S. aureus* iron limitation appears to stimulate biofilm formation. Other studies evidenced that high levels of iron can also compromise biofilm formation, and thus it is necessary to take into account the available amount of iron and establish equilibrium [228,231,232]. As such, it is possible to ascertain that both iron limitation and iron excess influence biofilm development.

Metals are also critical in the maintenance of the biofilm architecture, due to their involvement in the stability and cohesiveness of the extracellular matrix [233]. For instance, calcium and iron ions have been frequently emphasized for their cross-link properties among components of the biofilm matrix, helping in the maintenance of the matrix integrity [234,235]. On the other hand, some iron salts can be toxic for bacteria and disrupt preformed biofilms [231]. This proposes the two-edged sword role of metal chelators.

Taking into account that metals are critically implicated in bacterial adhesion, biofilm establishment and disruption, as well as their involvement in host infection by pathogens, targeting bacteria with metal-binding agents can provide a means for fighting biofilm infections. Several well-known synthetic chelators such as diethylenetriaminepentacetic acid, ethylenediaminetetraacetic acid, ethylenediamine-*N*,*N*9-diacetic acid, deferoxamine mesylate, 2,2′-dipyridyl, citrate and acetohydroxamic acid have been shown to interfere with biofilm formation and/or destabilize its structure, inducing inclusively in some cases the death of biofilm cells [233,236,237,238,239,240]. Some of these chelators are bacterial membrane-targeting compounds that have the ability to form complexes with metal ions. This results in metal removal from the lipopolysaccharides of the cell membranes and, consequently, in their disruption. Furthermore, chelating agents exert their effect on biofilm through the formation of complexes with metal ions of the extracellular matrix sequestering them, inducing biofilm dispersal [237].

Plant-derived compounds with chelating properties have been also found. Good examples of such compounds are polyphenols, phenolic acids and flavonoids, with the 6,7-dihydroxy iron chelation site being of particular interest inflavonoids [241,242,243]. Despite the great potential of phytochemicals to uptake metals, the study of their efficacy to treat biofilm-based infections continues to be scarce. Lin et al. [244] observed that an active ingredient of many medicinal plants belonging to the gallotannin class, 1,2,3,4,6-Penta-*O*-galloyl-b-d-glucopyranose (PGG), inhibits *S. aureus* biofilm formation. Also, they demonstrated that the antibiofilm activity of PGG is related to their ability to chelate iron. Extracts from cranberry juice impaired *E. coli* growth as well as the expression of genes associated with iron transport and the synthesis of metabolic enzymes, which was consistent with their capability to deplete iron. It is interesting to note that the biological effects attributed to cranberry have been mainly attributed to its anti-adhesive properties [245]. Based on the screening of several quinolines (a quinoline ring occurs in several plant compounds such as quinine alkaloids) it was possible to identify new compounds with potent inhibitory and dispersal activity against *S. aureus* and *S. epidermidis* biofilms. One of the possible mechanisms of the quinolines tested is metal-chelating, as they have a similar structure to nitroxoline, an antibiotic with recognized antimicrobial and antibiofilm activities, which was associated with the chelation of various metals [246]. In other work undertaken by Lee and collaborators [247], it was found that three natural anthraquinones (alizarin, purpurin and quinalizarin), among 560 compounds tested, efficiently inhibited *S. aureus* and *S. epidermidis* biofilm formation and the haemolytic activity of *S. aureus*. In the case of alizarin, one important aspect related to its antibiofilm effects was the ability to form complexes with calcium. A structure-activity relationship analysis revealed also that the two hydroxyl units at the C-1 and C-2 positions of anthraquinone play crucial roles in the mentioned activities.

The combined application of chelators with antimicrobial agents in clinical use is being evaluated and has revealed promising results [248,249,250]. An increased antibiofilm effect based on conjugates consisting of antibiotics and natural/synthetic siderophores, high-affinity iron-chelating compounds of low molecular weight, was observed [251]. Furthermore, other studies have been conducted to test the synergistic activity of chelating agents with other compounds, such as EPIs, providing promising results [236].

### 6.3. Phytochemicals as Biofilm Efflux Pump Inhibitors

As stated above, the expression of EPs has been reported as one of the mechanisms responsible for the tolerance of biofilms to antimicrobials [58,74]. The role of EPs in biofilm resistance has been shown for several biofilm-forming bacteria such as *P. aeruginosa*, *E. coli*, *Salmonella enterica* serovar Typhimurium, *S. aureus* and *Listeria monocytogenes* [252,253]. However, over the years this question was not so clear and the contradictory findings encountered suggest that the involvement of the EPs’ expression in biofilm-phenotype resistance can depend on several factors (e.g., biofilm physiology, experimental conditions and the strain) [254,255]. Currently, increasing evidence has elucidated that EP systems are not merely pumps involved in the transport of drugs or other toxic substances out of the cells but they have been also implicated in QS regulation [256] and the subsequent expression of genes responsible for virulence and biofilm formation [105,252,253,257,258]. Indeed, several studies demonstrated this linkage, pointing out the existence of the upregulation of genes encoding EPs during biofilm formation. For example, the expression of a novel EP (PA1874-1877) identified in *P. aeruginosa* is higher in biofilm cells than in planktonic cells [259]. Moreover, Pagès and co-workers [260] proposed that among all the known *E. coli* genes codifying for EPs, some may be specific for biofilms. *E. coli* mutants lacking the *emrD*, *emrE*, *emrK*, *acrD*, *acrE* and *mdtE* EP encoding genes displayed reduced biofilm formation ability compared with wild-type strains [261]. Similarly, *S. enterica* serovar Typhimurium strains, mutants in multidrug-resistant EPs, showed altered curli production which is an essential constituent of the extracellular polymeric substances (EPS) matrix, resulting in deficient biofilm formation [262]. Recently, in a study performed by Pantel et al. [263], the impact of EPs on biofilm formation, motility and virulence using an in vivo model of *C. elegans* was evaluated. They found that the overproduction of EPs favours the mentioned aspects.

It is worth noting that EPs can also negatively influence the potency of QSI [264]. Aybey et al. [265] verified that psychotropic drugs showing the ability to inhibit EPs also affect different QS-regulated behaviours. A report of Poole and colleagues [266] suggested that the MexAB-OprM efflux system is of crucial importance in *P. aeruginosa* survival under iron restrictions, as it is overexpressed under these circumstances. Furthermore, it was demonstrated that iron limitation has positive effects on the expression of both EPs (*ade*ABC) and QS genes (*lux*I/R) in *A. baumannii*, suggesting their regulatory role in these systems [267]. A surface protein named Bap, which is involved in *A. baumannii* biofilm formation (it helps in cell-cell adhesion and biofilm maturation), was also identified in strong biofilm formers in the described conditions [267].

Considering that EPs are highly active in bacterial biofilms, their use can be an attractive measure to control biofilm formation and/or reduce biofilm tolerance to antibiotics [268,269]. EPIs alone or combined with other compounds such as chelating agents and classical antibiotics could constitute a new antibiofilm strategy. Indeed, the combinatorial application of EPIs/chelators and antibiotics seems to be promising, as it has been demonstrated that these associations restore the potency of current antibiotics and reduce the rate of emergence of antibiotic-resistant variants [236]. A number of EPIs from synthetic and natural origins are already known and some examples were given previously. Some of them can also target biofilm formation [101,103,104,252].

Examples of EPIs from natural sources with antibiofilm activity comprise the alkaloid reserpine. It was found that this compound inhibited biofilm formation in clinical isolates of *K. pneumoniae* at low concentrations, which suggests that its effect can be due to EP inhibition [270]. Caffeoylquinic acids from *A. absinthium* were identified as an EPI [231]. Additionally, the authors demonstrated that the combined application of these compounds with ethidium bromide and moxifloxacin at sub-inhibitory concentrations promoted the inactivation of *S. aureus* and *E. faecalis* biofilms. Abouelhassan et al. [271] showed that gallic acid dramatically potentiates the antibacterial activities of several halogenated quinolines (up to 11 800-fold potentiation against *S. aureus*) against pathogenic bacteria, including drug-resistant clinical isolates, and found activity in biofilm eradication against a MRSA clinical isolate.

## 7. Concluding Remarks and Future Perspectives

A novel and promising approach to deal with multidrug resistance is to improve the clinical performance of various antibiotics by employing active molecules capable of restoring antibiotic susceptibility in MDR pathogens. The design of such s combination is a promising alternative that takes into account the scarcity of new and effective therapeutic antibacterials, especially against Gram-negative bacteria. Unfortunately, despite considerable effort, very few effective compounds have been obtained, and only one or two have come close to market [119].

Plants have been explored comprehensively as potential sources of EPIs and other antibiotic adjuvants. There have been numerous publications describing the isolation and purification of plant-derived antimicrobials. The known inhibitors, however, are mostly active against MDR pumps of Gram-positive species; efforts to find inhibitors of Gram-negative species have not been successful so far.

The widespread recognition that the majority of microorganisms are able to develop multicellular communities, impacting a myriad of environments with particular concern regarding infectious diseases, also increased the interest of the medical and scientific community in understanding the mechanisms underlying biofilm formation and resistance. Advances in the understanding of the biofilm formation and resistance mechanisms are significant. However, the progress on the development of strategies for their effective control is modest. Among the promising strategies, the use of phytochemicals has attracted significant attention, as they can be used as a source of new scaffolds for the development of novel therapeutic compounds with enhanced activity; they can also target bacteria using different mechanisms of current antibiotics. Another interesting aspect of the use of phytochemicals against biofilms is their ability to influence regulatory mechanisms involved in biofilm formation and maintenance by a non-bactericidal mode of action, and thus they do not impose selective pressure upon bacteria. However, caution in the application of these antivirulence approaches, such as the inhibition of QS pathways, is needed. In fact, they can favour or select for more virulent strains. In this sense, some studies have been conducted that have demonstrated that the development of resistance to QSI can occurs by several mechanisms (mutations in QS circuits, efflux pumps that can restrict the availability of QSI, inactivation or even modification of the target), although with less probability when compared to conventional antibiotics. Based on this evidence and due to the multiplicity of QS systems exhibited by some bacteria, the experts in this area are apprehensive and propose the use of QSI with a lower risk of developing resistance, including QSI uncompetitive and QSI with a broad range of activity [272,273]. Although it is unlikely that a single molecule will be able to act simultaneously in different QS receptors, as phytochemicals have a naturally distinctive multi-target mode of action, their combined application with other natural-based molecules, synthetic-based antimicrobials or enzymes can be helpful to extend the range of QSI. Furthermore, phytochemicals can be applied for drug repurposing as antibiotic potentiators helping to recycle old antibiotics by unknown mechanisms. In fact, an important missing piece from the studies of plant-derived antimicrobial/antibiofilm compounds is the understanding of their mode of action. As the precise nature of their working mechanisms is very important and useful in drug discovery processes, computer-based methodologies (e.g., molecular docking) could be used to predict the ligand-receptor binding/affinity. Moreover, the effort to discover new drugs from plants is threatened by the fact that this process is time-consuming and labourious, consisting mostly of the bioassay-guided isolation of different components of the extract and the successive identification of active compounds using numerous techniques. Therefore, new perspectives and methodologies, such as multivariate data analysis coupled to metabolomics, are promising for plant-based drug discovery. High-throughput screening can be also applied for the screening of a large amount of antimicrobials/antibiofilm compounds, therefore speeding up the development of new formulations [56].

Currently, the unique method that make the treatment of biofilm infections possible is the administration of several antimicrobial agents at high doses and during prolonged periods. Indeed, so far, there are no identified compounds with total efficacy against biofilms [130]. To surpass this drawback the combined application of several strategies will allow targeting different biofilm sites. For instance, combinatorial therapies based on physical (e.g., antimicrobial photodynamic therapy (APDT); electrical fields; surface modification), chemical (e.g., antimicrobials; disinfectants; plant extracts/phytochemicals; surfactants; metal chelators) or biological (e.g. bacteriophages; enzymes; antibodies) methods have been suggested for successfully fighting multidrug-resistant microorganisms and their biofilms [137].

## Figures and Tables

**Table 1:** Recent studies on QSIs identified from plants and their effect on QS-mediated mechanisms (survey from 2010 to 2016).

Source	Effect(s)	Bacteria	Reference(s)
**Plant Crude Extracts**			
Aqueous extracts from *Ananas comosus*, *Musa paradiciaca*, *Manilkara zapota* and *Ocimum sanctum*	-Inhibition of biofilm formation and virulence factor production (pyocyanin, staphylolytic protease and elastase); Reduction of *N*-Acyl homoserine lactones (AHLs)-mediated violacein production	*P. aeruginosa* PAO1; *Chromobacterium violaceum* CV12472	[156]
Extracts from *Prunus armeniaca, Prunella vulgaris, Nelumbo nucífera, Panax notoginseng, Punica granatum, Areca catechu* and *Imperata cylindrica*	-Inhibition of violacein production and swarming motility	*C. violaceum* CV026; *P. aeruginosa* PAO1	[157]
Methanolic extract from *Capparis spinosa* Linn.	-Inhibition of biofilm formation and motility (swimming and swarming); Decreases the biosurfactant and EPS production; Inhibition of violacein production	*P. aeruginosa* PAO1; *E. coli* ATCC 10536; *P. mirabilis* ATCC 7002; *Serratia marcescens* FJ584421; *C. violaceum* CV026/CV12472	[158]
Ethanolic extracts of Italian medicinal plants *Ballota nigra*, *Castanea sativa* and *Sambucus ebulus*	-Inhibition of δ-haemolysin production through of the interference with *agr* (accessory gene regulator) locus	MRSA (NRS385) clinical isolate	[159]
Methanolic, ethanolic, chloroformic, acetonic and aqueous extracts from *Salvadora persica*	-Inhibition of initial adhesion and biofilm formation; Interferes with QS regulators, *Streptococcus* OmpP and *Staphylococcus* Lux proteins	*S. mutans* cariogenic isolates	[160]
Crude extract and ethanolic fraction from *Emblica officinalis*	-Reduces cell adherence, cell-surface hydrophobicity, glucan synthesis and biofilm formation; Suppresses the expression of genes involved in biofilm formation; Obliterates biofilm structure	*S. mutans* MTCC 497	[161]
Extract of *Cymbopogan citratus* (lemongrass)	-Inhibition of biofilm formation; eradication of pre-formed biofilms	*S. aureus* NCIM 5022	[162]
Ethanolic extracts from *Hydrastis canadensis* L. (Ranunculaceae)	-Anti-QS activity by attenuation of signal transduction through the AgrCA component system; Inhibit toxin (alpha-toxin) production and prevents keratinocyte damage	*S. aureus* (CA-MRSA USA300 TCH1516, LAC, AH1263 and SA502A)	[163]
Methanolic extracts from *Achyranthes aspera* L. (Amaranthaceae)	-Inhibition of QS through of the interaction with OmpR QS regulator; Prevent glycosyltransferase (EPS synthesizing enzyme) expression; Inhibit biofilm formation	*S. mutans* cariogenic isolates	[164]
Hexane extract from *Amphypterygium adstringens*	-Inhibition of pyocyanin and rhamnolipid production; Decreases the elastolytic activity; Inhibition of violacein production	*P. aeruginosa* PA14; *C. violaceum* CV12472	[165]
Methanolic extracts from *Securinega suffruticosa*, *Angelica dahurica*, *Rodgersia podophylla*, *Viburnum carlesii*, *Nymphaea tetragona var.* *Angusta* and *Mallotus japonicus*	-Inhibition of swarming motility; Inhibition of violacein production	*P. aeruginosa* PAO1; *C. violaceum* CV12472	[166]
Methanolic extract rich in ellagic acid derivatives from *Terminalia chebula* Retz.	-Downregulation of *lasIR* and *rhlIR* genes expression; Attenuation of virulence factor production (pyocyanin, elastase, rhamnolipid and protease); Reduction of alginate production; Inhibition of biofilm formation and enhanced sensitivity of biofilm towards tobramycin; Enhanced survival of *Caenorhabditis elegans*	*P. aeruginosa* PAO1	[167]
*n*-hexane extract of *Dalbergia trichocarpa*	-Inhibition of biofilm formation, motility (swarming and twitching) and virulence factor production (pyocyanin, elastase and proteases); Increases the effectiveness of biofilm-encapsulated to tobramycin	*P. aeruginosa* PAO1	[168]
Ethyl acetate fraction of ethanol extract from *Syzygium cumini* L., *Pimentadioica* L., *Centella asiatica* L. and *Adenanthera pavonina* L.; Flavonoid fraction from leaves of *Psidium guajava* L.	-Inhibition of several QS-regulated phenotypes, namely: pyocyanin production, elastolytic and proteolytic activities, swarming motility and biofilm formation; Inhibition of violacein production	*P. aeruginosa* PAO1; *C. violaceum* CV026/CV12472	[169,170,171,172]
Polyphenol rich extract *from Rosa rugosa* tea	-Inhibition of swarming motility and biofilm formation; Inhibition of violacein production	*E. coli* K-12; *P. aeruginosa* PAO1; *C. violaceum* CV026/CV12472	[173]
Extracts of neotropical rainforest plants from *Meliaceae*, *Melastomataceae*, *Lepidobotryaceae*, and *Sapindaceae* families	-Biofilm formation and violacein production inhibition	*P. aeruginosa* PA14; *C. violaceum* CV12472	[174]
Extract from wheat bran	-Interferes with QS by degrading AHLs; Inhibition of biofilm formation; Eradication of pre-formed biofilms	*Pseudomonas fluorescens* (P3/pME6863 and P3/pME6000); *S. aureus* (BMA/FR/0.32/0074) clinical isolate	[175]
Extracts from *Chamaemelum nobile* (Chamomile)	-Inhibit biofilm formation, swarming motility	*P. aeruginosa* PAO1 and clinical isolates from different types of infections	[176]
Methanolic extract from *Kalanchoe blossfeldiana*	-Reduces virulence factors secretion (protease and pyoverdine) and cytokine formation in lipopolysaccharide-stimulated peripheral blood mononuclear cells; Inhibition of biofilm production	*P. aeruginosa* MTCC 2453	[177]
Ethanolic extract from *Amomum tsaoko*	-Inhibition of violacein production, swarming motility and biofilm formation	*C. violaceum* ATCC 12472; *S. aureus* ATCC 6538; *Salmonella enterica* serovar Typhimurium ATCC 50013; *P. aeruginosa* ATCC 9027	[178]
Methanolic extract from *Anethum graveolens* and one of its principle active, 3-*O*-methyl ellagic acid	-Downregulation of QS-regulated genes (*fimC, bsmA* and *flhD*) crucial for initial adhesion and motility; Reduction of biofilm formation and virulence factors (prodigiosin and protease) production	*S. marcescens* MG1/MG44 and clinical isolates	[179]
Methanolic extract from *Sygygium aromaticum*	-Inhibition of biofilm formation; Inhibition of EPS and pyocyanin production, proteolytic activity and swimming motility	*C. violaceum* ATCC 12472; *P. aeruginosa* clinical isolates	[180]
Methanolic extracts rich in ursene and oleanene derivatives (pentacyclic triterpenes) from *Castanea sativa* (European chestnut)	-Inhibition of haemolytic activity, harmful exotoxins (e.g., δ-toxin) production and biofilm formation (to a lesser extent) as results of the *agr*-mediated QS blockage; Attenuate skin abscesses in an in vivo animal model	*S. aureus* (AH408, SA502A, AH430, AH845, AH1263, LAC, AH1677, AH1747, AH1872, AH2759 and AH3052)	[181]
*n*-hexane and dichloromethane extracts of *Liriodendron hybrid* barks	-Inhibition of violacein production; Inhibition of biofilm formation	*C. violaceum* ATCC 12472/CV026; MRSA clinical isolates	[182]
Extract rich in polyphenols (orcinol, arabitol, apigenin, and usnic acid) from *Usnea longissimi* (Beard lichen)	-Inhibition of violacein production; Reduction of virulence factor secretion (acid production, ATPase, enolase, lactate dehydrogenase, protease, total exopolysaccharide content and glucosidase); Inhibition of biofilm formation; Improvement of the susceptibility to conventional antibiotics	*C. violaceum* CV12472; *S. mutans* MTCC 0497 clinical strain	[183]
Crude extract and methanolic fraction from Z*. officinale*	-Inhibition of biofilm formation; Reduce the insoluble glucan synthesis and sucrose-dependent adherence; Induce the dispersal of biofilm cells; Reduce caries development using an in vivo mouse model	*S. mutans* UA159	[184]
Extract rich in flavonoids (licoricone, glycyrin and glyzarin) from *Glycyrrhiza glabra*	-Reduce the production of QS-regulated virulence factors (e.g., motility, biofilm formation and production of antioxidant enzymes); Downregulation of the autoinducer (AI) synthase gene (*abaI*) expression	*A. baumannii* ATCC 19606 and ATCC 17978, and clinical isolates (M2, M2 (*abal*::Km), M2 (Pabal-*lac*Z) and C1-C4)	[185]
Methanolic extracts rich in tannin from *Phyllanthus emblica*, *Terminalia bellirica*, *Terminalia chebula*, *Punica granatum*, *Syzygium cumini*, and *Mangifera indica*	-β-lactamase inhibition as a result of the interference with *agr* expression; Inhibition of violacein production	*S. aureus agr*P3::*blaZ* RN6390 pRN8826; *C. violaceum* CV12472	[186]
Ethanolic extract from *Piper betle*	-Reduces swarming, swimming, and twitching; Inhibition of biofilm formation and pyocyanin production	*P. aeruginosa* PAO1	[187]
Phenolic extract from *Rubus rosaefolius* (wild strawberry)	-Inhibition of several QS-regulated phenotypes, namely: violacein production, swarming motility and biofilm formation	*C. violaceum* ATCC 6357; *S. marcescens* UFOP-001; *Aeromonas hydrophila* IOC/FDA110	[188]
Ethanol solution extract rich in evodiamine, rutaecarpine and evocarpine from *Euodia ruticarpa*	-Inhibition of AI-2 production; Inhibition of cell adhesion and biofilm formation	*Campylobacter jejuni*	[189]
**Plant pure compounds**			
Epigallocatechin gallate from green tea	-Inhibition of biofilm formation and swarming motility; Synergistic activity with ciprofloxacin in the treatment of biofilm infections	*P. aeruginosa* PAO1	[190]
Catechin and naringenin from *Combretum albiflorum*	-Interfere with the pyocyanin and elastase production; Affect the AIs perception; Biofilm formation inhibition	*P. aeruginosa* PAO1	[191]
Catechins with a galloyl moiety (e.g., epichatechin gallate and epigallocatechin gallate)	-Affect AI-2 and inhibit biofilm formation	*Eikenella corrodens* 1073	[192]
Saponins, ginsenosides, and polysaccharides fom *Panax ginseng*	-Suppression of the production of LasA and LasB; Downregulation of AHLs synthesis; Clearance of pulmonary infections in animal studies by biofilm disruption	*P. aeruginosa* PAO1 and its isogenic mucoid variant (PAO*mucA22*)	[193,194]
Baicalin hydrate, cinnamaldehyde and hamamelitannin	-Increase biofilm susceptibility to treatment with antibiotics (e.g., tobramycin, clindamycin, vancomycin); Enhance the survival of infected *C. elegans* and *Galleria mellonella*; Reduce the microbial load in the lungs of infected BALB/c mice	*P. aeruginosa* (PAO1, ATCC 9027, MH340 and MH710); *Burkholderia cenocepacia* (LMG 16656, LMG 16659 and LMG 18828); *S. aureus* (LMG 10147 and Mu50–MRSA) and clinical isolates (CS1)	[195]
Chrysophanol, nodakenetin, shikonin and emodin from tradicional Chinese herbs (*Rheum officinale* Baill, *Peucedanum decursivum* (Miq). Maxim, *Lithospermum erythrorhizon* Sieb, *Rheum palmatum* L.)	-Inhibition of biofilm formation; Potentiation of the ampicillin activity; Proteolysis of the QS signal receptor TraR	*Stenotrophomonas maltophilia* GIMT1.118; *P. aeruginosa* PAO1; *E. coli* BL21(DE3)	[196]
Allicin and ajoene from *Allium sativum*	-Reduction of QS-controlled virulence genes expression; Attenuation of the rhamnolipid production; Synergistic activity with tobramycin on biofilms; Cessation of the polymorphonuclear leukocytes lytic necrosis; Enable the clearance of pulmonary infections in mouse models	*P. aeruginosa* PAO1	[143,197]
Iberin from *Armoracia rusticana* (horseradish)	-Blockage of the QS-regulated genes expression by targeting LasIR and RhlIR QS networks; Downregulation of rhamnolipid production	*P. aeruginosa* PAO1	[146]
Methyl eugenol from *Cuminum cyminum*	-Inhibition of biofilm formation, motility (swimming and swarming) and EPS production	*P. aeruginosa* PAO1; *P. mirabilis* ATCC 7002; *S. marcescens* FJ584421	[198]
Rosmarinic acid, naringin, chlorogenic acid, morin and mangiferin	-Inhibition of biofilm formation and virulence factor production (protease, elastase and haemolysin)	*P. aeruginosa* PAO1; *P. aeruginosa* AS1 and AS2	[199]
Curcumin from *Curcuma longa* L.	-Inhibition of biofilm formation and attenuation of QS-dependent factors (exopolysaccharide and alginate production); Inhibition of swimming and swarming motility; Biofilm susceptibility enhancement to antibiotics; Enhanced survival rate of *Artemia nauplii*	*E. coli* ATCC 10536; *P. aeruginosa* PAO1; *P. mirabilis* ATCC 7002; *S. marcescens* FJ584421; *Vibrio harveyi* MTCC 3438, *Vibrio parahaemolyticus* ATCC 17802 and *Vibrio vulnificus* MTCC 1145	[200,201]
Glycosylflavonoids (chlorogenic acid, isoorientin, orientin, isovitexin, vitexin, and rutin) from *Cecropia pachystachya* Trécul	-Inhibition of violacein production; Inhibition of bioluminescence production	*C. violaceum* ATCC 31532; *E. coli* pSB403	[202]
Salicylic acid	-Inhibit swimming motility; Dual-species biofilms enhancement to a second exposure to salicylic acid	*Bacillus cereus* isolated from a disinfectant solution; *P. fluorescens* ATCC 13525	[203]
Salicylic acid, tannic acid and trans-cinnamaldehyde	-Inhibition of AHLs and pyocyanin production	*P. aeruginosa* PAO1	[204]
[6]-gingerol, [6]-shogaol and zingerone from Z*ingiber officinale* Roscoe	-Inhibition of biofilm formation, violacein and pyocyanin production	*C. violaceum* MTCC 2656; *P. aeruginosa* MTCC 2297/PA14	[205,206]
Zingerone from ginger root	Decreases swimming, swarming and twitching motility; Reduces biofilm-forming capacity; Interferes with the production of virulence factors including rhamnolipid, elastase, protease, pyocyanin; Improves the antibiofilm efficacy of ciprofloxacin	*P. aeruginosa* PAO1	[207,208]
Sesquiterpenoid viridiflorol and triterpenoids, ursolic and betulinic acids, from the liverwort *Lepidozia chordulifera*	-Inhibition of biofilm formation and elastolytic activity	*P. aeruginosa* ATCC27853; *S. aureus* ATTC6538	[209]
Malvidin of methanolic extract from *Syzygium cumini*	-Inhibition of violacein production, EPS synthesis and biofilm formation; Potentiation of the susceptibility to conventional antibiotics	*C. violaceum* CV026/MTCC 2656; *K. pneumoniae* PUFST23	[210]
Hamamelitannin	-Increases the in vitro biofilm susceptibility to vancomycin treatment through the TraP receptor by affecting cell wall biosynthesis (peptidoglycan) and extracellular DNA release; Increases the in vivo susceptibility to antibiotic treatment using *C. elegans* and mouse (mammary gland infection) models	MRSA Mu50	[211]
**Essential oils and components**			
Clove essential oil	-Inhibition of LasB, total protease, chitinase, pyocyanin and exopolysaccharide production; Swimming motility and biofilm formation reduction	*P. aeruginosa* PAO1; *A. hydrophila* WAF-38	[212]
Essential oil from *Murraya koenigii*	-Inhibition of violacein production; Biofilm formation inhibition; Reduces cell adhesion, metabolic activity and EPS production; Prevents biofilm maturation	*C. violaceum* CV026/CV12472; *P. aeruginosa* PAO1	[213]
Cinnamon oil	-Inhibits biofilm formation and virulence factors production (pyocyanin, rhamnolipid, and protease); Reduces alginate and EPS production, and swarming motility	*P. aeruginosa* PAO1	[214]
*Mentha piperita* essential oil (peppermint) and menthol	-Inhibition of violacein production; Biofilm formation, EPS production and swarming motility inhibition; Affect QS regulate virulence factors production (elastase, total protease, pyocyanin and chitinase); Interference with *las* and *pqs* QS systems; Enhanced survival of *C. elegans*	*C. violaceum* CV026; *P.aeruginosa*; *A. hydrophila*; *E. coli* (MG4/pKDT17 and pEAL08-2)	[215]
Clove bud oil	-Attenuation of extracellular DNA, exopolysaccharides and pigment production; Decreases the transcription of *pqsA* gene; Biofilm inhibition and dispersal	*P. aeruginosa* PAO1	[216]
Eugenol	-Inhibition of violacein production, elastase, pyocyanin and biofilm formation; Interference with *las* and *pqs* QS systems	*C. violaceum* CV026; *P. aeruginosa* PAO1/PAO-MW1; *E. coli* (MG4/pKDT17 and pEAL08-2)	[217]
Carvacrol	-Inhibition of biofilm formation; Reduction of the expression of *cviI* gene, production of violacein and chitinase activity	*C. violaceum* ATCC 12472; *S. enterica* serovar Typhimurium DT104; *S. aureus* 0074	[218]

## Abbreviations

The following abbreviations are used in this manuscript:

ABC	ATP binding cassette
*agr*	accessory gene regulator
AHLs	*N*-Acyl homoserine lactones
c-di-GMP	Cyclic diguanosine monophosphate
EPS	Extracellular polymeric substances
ESBLs	Extended-spectrum β-lactamases
EPs	Efflux pumps
EPIs	Efflux pump inhibitors
EPS	Extracellular polymeric substances
FDA	Food and Drug Administration
MF	Major facilitator
MIC	Minimum inhibitory concentration
MATE	Multidrug and toxic efflux
MDR	Multidrug resistant
MRSA	Methicillin-resistant *S. aureus*
PGG	1,2,3,4,6-Penta-*O*-galloyl-b-d-glucopyranose
QS	Quorum sensing
QQ	Quorum-quenching
QSI	QS inhibitors
RND	Resistance-nodulation-division
SMR	Small multidrug resistance
